# Rapid development of naked malting barley germplasm through targeted mutagenesis

**DOI:** 10.1007/s11032-025-01553-5

**Published:** 2025-03-07

**Authors:** Hiroshi Hisano, Hiroaki Sakai, Mika Hamaoka, Hiromi Munemori, Fumitaka Abe, Brigid Meints, Kazuhiro Sato, Patrick M. Hayes

**Affiliations:** 1 Institute of Plant Science and Resources, Okayama University, 2-20-1 Chuo, Kurashiki, Okayama 710-0046 Japan; 2 Research Center for Advanced Analysis, National Agriculture and Food Research Organization, Tsukuba, Japan; 3 Institute of Crop Science, National Agriculture and Food Research Organization, Tsukuba, Japan; 4 Department Crop and Soil Science, Oregon State University, Corvallis, OR USA; 5 Faculty of Agriculture, Setsunan University, Hirakata, Japan; 6 Kazusa DNA Research Institute, Kisarazu, Japan

**Keywords:** *Hordeum vulgare*, Covered (hulled), Naked (hull-less), Genome editing, CRISPR/Cas9, Transformation amenability

## Abstract

Covered barley (*Hordeum vulgare*) has historically been preferred for malting, as the husk in this plant protects the embryo during harvest and acts as a filter during brewing. Naked barley, which is typically used as food, has the potential to be used in brewing due to recent technical advances, but the grains contain higher levels of β-glucan and polyphenols, which are undesirable in brewing. Introducing the naked trait into brewing cultivars through crossing is time-consuming due to the need to eliminate these undesirable traits. In this study, we rapidly developed naked barley that is potentially suitable for malting by introducing targeted mutations into *Nudum* (*NUD*) using CRISPR/Cas9-mediated targeted mutagenesis. The doubled haploid line ‘DH120366’, which was used as the parental line, was derived from a cross between two covered malting barley cultivars. We generated CRISPR/Cas9-mediated targeted mutagenized barley harboring mutations in *NUD* via *Agrobacterium tumefaciens*-mediated transformation and confirmed the presence of mosaic mutations in one individual from among 16 T_0_ transformants. We sowed T_1_ grains exhibiting the naked trait and sequenced the *NUD* gene in these T_1_ seedlings, identifying two types of mutations. Shotgun high-throughput whole-genome sequencing confirmed the absence of the transgene in at least one *nud* mutant line following *k*-mer-based analysis. Cultivation in a closed growth chamber revealed no significant differences in agronomic traits between the *nud* mutants and the wild type. This study demonstrates the feasibility of rapidly developing naked barley with potential use for malting and brewing by targeting only *NUD* via targeted mutagenesis.

**Supplementary Information:**

The online version contains supplementary material available at 10.1007/s11032-025-01553-5.

## Introduction

Barley (*Hordeum vulgare*) is a major cereal crop, ranking fourth in annual worldwide production after maize (*Zea mays*), wheat (*Triticum aestivum*), and rice (*Oryza sativa*). Barley serves multiple purposes, including as a food, for malt production, and for livestock feed. There are two types of barley caryopses: covered (hulled) and naked (hull-less). Throughout this report, we will use the preferred covered/naked nomenclature. Covered barley has traditionally been used for brewing because the husk protects the embryo from mechanical damage during harvesting and acts as a filter during the brewing process. However, advancements in high-performance filtering technology, such as mash filters, have made the husks of covered barley less necessary. Additionally, naked barley has the potential to have higher levels of malt extract than covered barley, which is desirable to brewers and distillers. Moreover, since barley husks contain unwanted flavor components like tannins, brewing or distilling with naked barley could result in beer or whisky with fewer undesirable flavors and less waste (Meints et al. 2021). Despite these potential benefits, limited resources have been directed to breeding naked barley for malt end-uses. Most breeding efforts have been targeted on food end-uses, as naked barley primarily serves as a staple food in Eastern Asia (Dickin et al. 2011; Lister and Jones 2013; Meints et al. 2021). Furthermore, since naked barley potentially contains higher levels of β-glucans and other water-soluble polysaccharides with positive health effects compared to covered barely, naked barley has recently attracted renewed interest for use as a food source in Western countries (Wood 2004).

During crop breeding, crossing cultivars with different genetic backgrounds poses challenges, as eliminating unwanted traits from one parent that are transferred to the progeny is time-consuming. Barley cultivars have been selectively bred for specific purposes, resulting in distinct genetic backgrounds characterized by the appropriate traits. For instance, barley cultivars bred for brewing exhibit malting-friendly germination and saccharification traits, such as uniform germination and saccharification performance, that are unnecessary for barley cultivars used as food sources. Conversely, polysaccharides such as β-glucan and arabinoxylan, which are beneficial for food use, are problematic in brewing because they cause haziness of the wort (brewing liquid). Consequently, the genetic differences between malting barley and food barley cultivars have limited the crossing of these types of barley, despite the potential for efficient cultivar development.

Targeted mutagenesis (commonly referred to as genome editing) is a powerful tool for mutant breeding. This technique allows mutations to be introduced precisely into target genes with minimal consequences to the overall genetic background. Over the past decade, this technology has led to significant advancements in plant science and breeding. Among the various targeted mutagenesis techniques, the clustered regularly interspaced short palindromic repeats (CRISPR)/CRISPR-associated nuclease 9 (Cas9) system stands out for its versatility and ease of use (Hisano et al. 2021).

The *Nudum* (*NUD*) gene, located on barley chromosome 7H (Taketa et al. 2004), controls the covered/naked caryopsis phenotype. *NUD* encodes an ethylene-responsive transcription factor that shares similarity with the WIN1/SHN1 transcription factor, which plays a role in lipid biosynthesis in Arabidopsis (*Arabidopsis thaliana*). In covered barley, the caryopsis and the lemma are both covered with lipids produced via NUD function, leading to their adherence (Taketa et al. 2008). Conversely, loss of NUD function results in easily dehusked and naked barley. The CRISPR/Cas9 system has been successfully adapted for knocking out *NUD* in barley, primarily thanks to the ease of scoring the mutant phenotype visually (Gasparis et al. 2018; Gerasimova et al. 2020). Gasparis et al. (2018) developed an efficient method using the *NUD* and *cytokinin oxidase/dehydrogenase* (*CKX*) genes. Overall, stable targeted mutagenesis in barley relies on *Agrobacterium tumefaciens* (*Rhizobium radiobacter*)-mediated gene transfer into the barley genome.

Our research group previously identified so-called *Transformation amenability* (*TFA*) loci and proposed a *TFA*-based selection system for developing transformable barley lines (Hisano and Sato 2016). In this system, the *TFA* loci located on chromosomes 2H and 3H are replaced with alleles from the malting barley cultivar ‘Golden Promise’, which can be transformed at acceptable rates, resulting in transformation-amenable individuals. To test the efficacy of this approach, we generated the Oregon Promise mapping population by crossing Golden Promise with ‘Full Pint’, which is recalcitrant to genetic transformation. Doubled haploid progeny carrying the *TFA* alleles from Golden Promise were amenable to transformation, underscoring the usefulness of this approach (Hisano et al. 2017). This *TFA*-based system opens the door to general transformation and targeted mutagenesis, allowing desirable lines with good agronomic traits to be directly bred. Using Golden Promise as a recipient, our research group has also achieved successful targeted mutagenesis of the grain dormancy genes *QTL for seed dormancy 1* (*Qsd1*) and *Qsd2*, the phosphate transporter gene *SULTR-like phosphorus distribution transporter* (*SPDT*), and *Mildew resistance locus O* (*MLO*) (Hisano et al. 2022; Gu et al. 2022; Koide et al. 2023). These two techniques, *TFA*-based selection and CRISPR/Cas9-based targeted mutagenesis, make it possible to introduce precise mutations into one or more genes within a cultivar or background of interest, allowing for trait modification without altering the genetic background beyond the desired mutations.

In this study, we employed the CRISPR/Cas9 system to generate naked barley from malting barley by disrupting the *NUD* gene. We obtained two mutant alleles from a single transformation event. We discuss critical issues for consideration before this cultivar can be released for cultivation, such as checking for the presence or absence of the transgene by PCR or high-throughput sequencing, as well as potential off-target sites. Analysis of various growth parameters and grain germination rate highlights the potential utility of these mutants.

## Materials and methods

### Plant materials

The doubled haploid barley line ‘DH120366’ from the Oregon Promise mapping population was chosen for this study. This population was derived from a cross between malting barley cultivars Golden Promise and Full Pint (Herb et al. 2017). DH120366 had an attractive malting quality profile in micro-malting tests (Table S1) and is amenable to transformation (Hisano et al. 2017). For plant transformation, DH120366 plants were initially grown under a 12-h light/12-h dark photoperiod and a 15°C/13°C (day/night) cycle for 8 weeks, followed by a 16-h light/8-h dark photoperiod and a 16°C/13°C (day/night) cycle until immature embryos were harvested.

### Targeted mutagenesis using the CRISPR/Cas9 system

To create knockout barley lines targeting *NUD* (HORVU.MOREX.r3.7HG0719680.1), ten single guide RNAs (sgRNAs) were designed (Table S2) using the online tool CRISPRDB (https://crisprdb.org/custom.html; accessed on November 18, 2024, Wong et al. 2015). The vectors pHvU3-21 and pZH_gYSA_PubiMMCas9 were used to express the sgRNAs and *Cas9* driven by the *HvU3* promoter and maize *Ubiquitin* promoter, respectively (Figure S1a). Vector construction followed a previous protocol (Gu et al. 2022). The complementary oligo DNAs shown in Table S2 were annealed and cloned into the *Bbs*I digestion site of pHvU3-21. The expression cassette of sgRNA in pHvU3-21 was then excised with *Pac*I and *Asc*I and introduced into the binary vector pZH_gYSA_PubiMMCas9. The resulting constructs (pZH-HvU3-NUDgRNA) were introduced into *Agrobacterium* strain AGL1 and used for targeted mutagenesis of barley via *Agrobacterium*-mediated transformation as described by Hisano et al. (2017). The genomic region targeted by each sgRNA was amplified by PCR and subjected to Sanger sequencing using specific primer pairs (NUD-check-F3: 5’-GAGAATCTCGCTCGCTCTG-3’ and NUD-check-R3: 5’-GATCTGTGACAGGCTGCTG-3’, Table S3). DNA extraction and PCR conditions were described by Hisano et al. (2022) and Hisano and Sato (2016), respectively.

### Generation of transgene-free mutant plants

All mutant plants were backcrossed twice with DH120366. Genomic DNA was extracted from approximately 200 mg of fresh leaves harvested from T_1_, F_1_, and BC_1_F_1_ plants (Figure S2) using a DNeasy Plant Mini Kit (QIAGEN) following the manufacturer’s protocol. PCR was performed for initial transgene detection using primer pairs specific to the hygromycin phosphotransferase gene *HPT* and *Cas9* to identify transgene-free plants. Direct Sanger sequencing with NUD-check-F3/R3 primer pairs was conducted to select mutant individuals.

### Analysis of transgenes and potential off-target sites

Genomic DNA sequencing libraries were prepared using an MGIEasy FS PCR-free DNA Library Prep Set (MGI Tech) with 1,000 ng of genomic DNA extracted from each plant using a DNeasy Plant Mini Kit (QIAGEN). Sequencing was performed using the DNBSEQ-G400RS platform with the corresponding high-throughput sequencing set (MGI Tech) as paired-end 150-bp reads. The *k*-mer-based method (Itoh et al. 2020) was applied using the DNA sequence of the pZH-HvU3-NUDgRNA8 construct as a template.

To assess off-target mutations, genomic regions with potential off-target effects were identified by BLAST search using the target site sequence as a query; these regions were then amplified by PCR, and the resulting amplicons were subjected to Sanger sequencing. For the BLAST search, the sgRNA target site (5’-GGCTGCGCGGGCGTACGATGNGG-3’) and its surrounding sequence (5’-CGGCGGAGGAGGCTGCGCGGGCGTACGATGAGGCTGCCATCCT-3’) in the *NUD* gene were used as a query against the barley genome reference databases “Barley pan-genome Golden Promise v1.0 pseudomolecules” (Schreiber et al. 2020) and “Barley Morex v3 pseudomolecules” (Mascher et al. 2021) with the advanced parameters: “-task blastn -evalue 100” at the GrainGenes BLAST server (https://wheat.pw.usda.gov/blast/). Additionally, Sanger sequencing of PCR amplicons was conducted using site-specific primer pairs (Table S3) for both the wild-type copy and each mutant.

### Evaluation of agronomic traits in the mutants

Control (wild type) and mutant plants (T_2_) were grown under a 16-h light/8-h dark photoperiod and a 16°C/13°C (day/night) cycle in a closed growth chamber (Figures S2 and S3). Culm length, thousand-grain weight, and hectoliter grain weight were measured for all plants at maturity. The grain germination rate was measured as described by Hisano et al. (2022). Briefly, harvested spikes were dried at 25°C for 10 days in a drying cabinet and stored at − 20°C. All spikes were hand-threshed simultaneously, and grains (T_3_) were after-ripened at 25°C and 10–15% relative humidity for 4–5 weeks. Each germination test was conducted using 50 grains per biological replication on filter paper (ADVANTEC, Japan) soaked in distilled water in 90-mm diameter disposable dishes with four replications at 25°C. The germination rate was calculated by counting seedlings with primary shoots and roots that had elongated to > 5 mm.

## Results

### Generation of naked barley plants

**Fig. 1:**
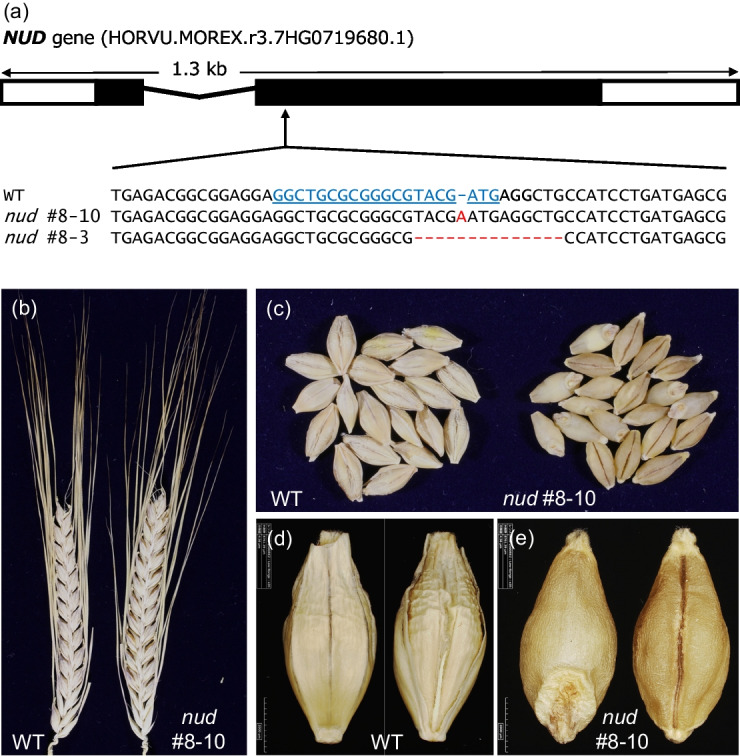
Genotypes and phenotypes of *nud* barley plants generated by CRISPR/Cas9-mediated targeted mutagenesis. **a** Structure of the *NUD* gene (HORVU.MOREX.r3.7HG0719680.1) and partial sequence alignment of the *NUD* gene in wild-type DH120366 (WT) and two *nud* mutant lines. Red letters and hyphens indicate insertion and deletion, respectively. Underlined blue letters represent the target sequence of single guide RNA8 (sgRNA8; see Table S2), and bold letters denote the protospacer-adjacent motif (PAM). **b, c** Representative phenotypes of WT and *nud* spikes and grains. Photographs show spikes** (b)** and grains** (c)**. **(d, e)** Dorsal view (left) and ventral view (right) of WT** (d)** and *nud* **(e)** barley grains

Our goal was to knock out *NUD* using the CRISPR/Cas9 system to develop naked lines from a malting barley. Accordingly, we designed 10 independent single guide RNAs (sgRNAs), each targeting a different position of the *NUD* coding region (Figure S1b). Following the transformation of immature embryos with the 10 resulting targeted mutagenesis constructs, we obtained 16 hygromycin-resistant plants: nine plants for sgRNA1, one plant for sgRNA4, four plants for sgRNA8, and two plants for sgRNA10. We sequenced PCR amplicons covering each corresponding target site by Sanger sequencing and identified one plant, referred to as ‘*nud* #8’, derived from the construct harboring sgRNA8. This *nud* #8 plant carried a mutation in the *NUD* target site. No clear mutations were detected in other plants. We cultivated this T_0_ plant in a closed growth chamber and harvested its T_1_ caryopses, some of which displayed a naked barley phenotype. We then selected 10 naked caryopses and 10 covered caryopses and planted them to obtain T_1_ seedlings. We extracted genomic DNA from these plants, subjected it to PCR, and performed Sanger sequencing to assess the presence of mutations in *NUD*. Some plants contained one or two mutations in *NUD*, while other plants had the wild-type genotype. One plant was homozygous for a 1-bp insertion (*nud* #8–10); one was homozygous for a 13-bp deletion (*nud* #8–3); one was heterozygous for the 13-bp deletion, carrying the wild-type sequence in the other copy of the gene (*nud* #8–8); and one exhibited a mosaic mutation (*nud* #8–4) (Fig. 1a, Figure S1c, Table S4). One naked caryopsis failed to germinate (*nud* #8–5). Importantly, none of the ten seedlings derived from the covered caryopses (from *nud* #8–11 to #8–20) harbored a mutation in *NUD*, in agreement with their phenotype (Table S4).

To assess the presence/absence of the transgene, we performed PCR with a primer pair specific for the hygromycin phosphotransferase gene (*HPT*). Agarose gel electrophoresis of the PCR products indicated that the T_1_ seedlings *nud* #8–3 and #8–8 were positive for the transgene, while the T_1_ seedling *nud* #8–10 was negative (Table S4). We focused on *nud* #8–3 and #8–10 seedlings for further analysis, as they were homozygous for a mutation in *NUD*. To confirm the absence of a transgene copy, we crossed the T_1_ plants *nud* #8–3 and #8–10 with wild-type DH120366 and genotyped the F_1_ generation by PCR using *HPT*- and *Cas9*-specific primers. The F_1_ plant *nud* #8–10 × DH120366 had no detectable transgene based on the absence of a PCR product in all 26 F_1_ plants tested. By contrast, all 28 F_1_ individuals derived from the *nud* #8–3 × DH120366 cross carried a transgene. We randomly chose two F_1_ individuals (*nud* #8–3–7 and #8–3–10) and crossed them to DH120366 (Figure S2). Notably, we identified two transgene-free plants heterozygous for their respective *nud* mutation (#8–3–7–8 and #8–3–10–5, as shown in Table S5) among 31 BC_1_F_1_ plants, which we subsequently subjected to high-throughput sequencing. The naked barley phenotype was observed in the F_2_ (for *nud* #8–10) and BC_1_F_2_ (for *nud* 8–3) generations (Fig. 1). Importantly, we observed no discernible differences in spike morphology or grain size between the wild type and F_2_ and BC_1_F_2_ plants with the naked phenotype (Fig. 1b–e).

### High-throughput sequencing to detect the transgene

We conducted high-throughput sequencing using total genomic DNA extracted from DH120366, *nud* #8–3 (T_1_), #8–10 (F_1_), #8–3–7–8, and #8–3–10–5 (BC_1_F_1_) to determine the presence or absence of the transgene for targeted mutagenesis. The sequencing data are summarized in Table S6. We obtained 388.7 and 434.1 million reads, with total lengths of 116.8 Gb and 134.6 Gb, respectively, from DH120366 (used as a negative control) and *nud* #8–3 (used as a positive control). For the mutant plants *nud* #8–3–7–8, #8–3–10–5, and #8–10, we obtained 337.1–393.2 million reads, covering a total length of 120.3–138.6 Gb (Table S6).

**Fig. 2:**
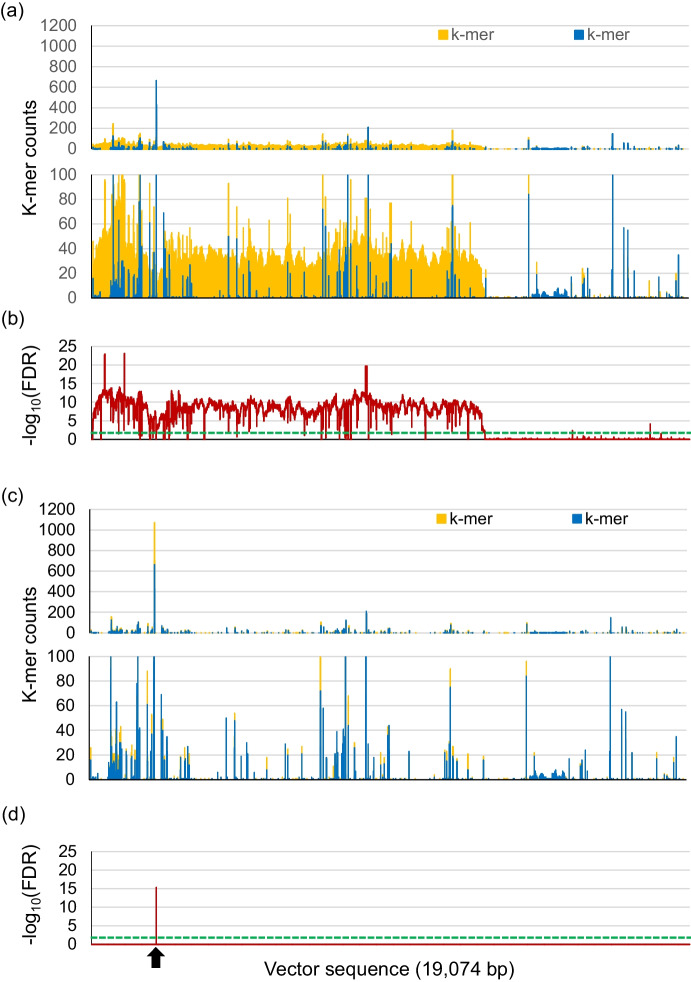
Transgene detection in *nud* barley plants using the *k*-mer-based method. **a, c** Number of reads counted by the *k*-mer-based method, and **(b, d)** false discovery rate (FDR)-adjusted *p-*values for potential transgene detection. The *x*-axis represents nucleotide positions covering the vector sequence from 1 bp to 19,074 bp, with the T-DNA region spanning 1 bp to 12,584 bp. Top, overall views; bottom, magnified views. Blue indicates read counts for the wild-type control, and yellow represents** (a)** *nud* #8–3 with a transgene and **(c)** *nud* #8–10 without a transgene. The solid red and dashed green lines correspond to the FDR-adjusted *p*-values as “ − log_10_(FDR)” and the 1% significance threshold, respectively, for** (a)** *nud* #8–3 and **(c)** *nud* #8–10. Black arrow indicates the position from 2,069 bp to 2,089 bp

To detect the transgene, we employed the *k*-mer-based method, where 20-bp segments extracted from all short reads were compared with the sequence of the construct pZH-HvU3-NUDgRNA8 used for transformation (Itoh et al. 2020). Figure 2 shows the *k*-mer counts and the false discovery rate (FDR)-adjusted *p*-values (expressed as − log_10_[FDR]) at each position along the construct sequence. In *nud* #8–3, *k*-mer counts were consistently abundant in the T-DNA region from 1 bp to 12,584 bp, whereas DH120366 and *nud* #8–10 had only sporadic *k*-mer counts along the same sequence (Fig. 2a and 2c). The average *k*-mer counts were 21.9 (*nud* #8–3), 1.0 (DH120366), and 0.7 (*nud* #8–10) (Table 1). Notably, the two BC_1_F_1_ individuals *nud* #8–3–7–8 and #8–3–10–5, resulting from a cross between *nud* #8–3 and DH120366, exhibited a similar low *k*-mer count (0.8) to *nud* #8–10. Focusing on the T-DNA region of the construct, *nud* #8–3 had an average *k*-mer count of 33.1, while the other samples had average *k*-mer counts of 0.8 (DH120366) and 1.1 (*nud* #8–3–7–8 and #8–3–10–5). The sequence outside the T-DNA yielded *k*-mer counts ranging from 0.1 to 0.5 across all samples. Notably, DH120366 had the highest *k*-mer counts (664 and 431) in the C-rich sequence 5’-CCTCCCCCCCCCCCCCTCTCT-3’ along the region from 2069 to 2089 bp (Fig. 2a).

**Table 1:** Summary of average mapped *k*-mers

		Average (counts/position)		
Sample	Note	T-DNA	non-T-DNA	Total average
DH120366	Negative control	0.8	0.5	0.7
*nud* #8–3	Positive control (T_1_)	33.1	0.1	21.9
*nud* #8–3–7–8	BC_1_F_1_	1.1	0.1	0.8
*nud* #8–3–10–5	BC_1_F_1_	1.1	0.1	0.8
*nud* #8–10	F_1_	1.0	0.1	0.7

In addition to the *k*-mer analysis described above, we calculated the FDR-adjusted *p*-values (as -log_10_[FDR]) in pairwise comparisons between each mutant plant and the wild type to assess the probability of transgene presence, with high FDR-adjusted *p*-values indicating the possibility of transgene presences. The FDR-adjusted *p-*values for *nud* #8*–*3 compared to DH120366 were significant (*p* < 0.01) over most of the T-DNA region, confirming the presence of a transgene in *nud* #8–3 (Fig. 2b). By contrast, *nud* #8–10 had significant FDR-adjusted *p*-values only along a specific C-rich region (from 2,069 bp to 2,089 bp, Fig. 2d). Notably, *k*-mers around this C-rich region were detected in all samples, including DH120366, suggesting a false positive signal. Additionally, *nud* #8–3–7–8 and #8–3–10–5 displayed one and two small regions with a significant FDR-adjusted *p*-value, respectively, that were distinct from the C-rich region described above (Figure S4b and S4d). These small regions with high *k*-mer counts with high FDR-adjusted *p*-values suggest that 20–21-bp transgene fragments remained in these plants, as no *k*-mers mapped to these regions in DH120366.

### Potential off-target sites

We conducted a BLAST search against the barley reference genome using the sequence targeted by sgRNA8, including the protospacer-adjacent motif (PAM), with the parameters “-task blastn -evalue 0.1”. This analysis yielded only one genomic region with a hit, corresponding to the *HvNUD* locus on chromosome 7H. However, when we lowered the standards for the BLAST search using the parameters “-task blastn -evalue 100,” we identified six more genomic regions matching the target site with no more than three mismatches. These regions were distributed across chromosomes 2H (one site), 6H (two sites), and 7H (three sites) (Table S3). To test the possibility that mutations were inserted at these potential off-target sites, we sequenced PCR products encompassing each site via Sanger sequencing using genomic DNA from DH120366, *nud* #8–3 (T_1_), #8–3–7–8 (BC_1_F_1_), #8–3–10–5 (BC_1_F_1_), and #8–10 (F_1_) plants. We did not detect off-target mutations in any of the mutants (see Figure S5).

### Evaluation of agronomic traits in the *nud *mutants

To assess agronomic traits, we simultaneously cultivated wild-type DH120366 and mutant plants (T_2_ and T_3 _*nud* #8–3 and #8–10) in a closed growth chamber. At maturity, the mean culm length was 85.0 cm for *nud* #8–3 and 84.2 cm for *nud* #8–10 compared to 84.1 cm for the wild type (Table 2). The thousand-grain weight did not differ significantly among the three genotypes, although it was lower in the mutants due to the absence of hulls: 34.20 g for *nud* #8–3, 33.05 g for *nud* #8–10, and 35.56 g for the wild type (Table 2). The hectoliter mass of both mutant lines (*nud* #8–3, 80.01 kg/hL; *nud* #8–10, 76.85 kg/hL) was significantly greater than that of DH120366 (64.60 kg/hL), as expected, due to the absence of hulls.

**Table 2:** Culm length, thousand-grain weight, and hectoliter grain weight of the wild type and *nud* mutants

	Culm length				Thousand-grain weight				Hectoliter grain weight			
Sample	*n*	Average (cm)	SD		*n*	Average (g)	SD		*n*	Average (kg/hL)	SD	
DH120366	25	84.06	2.91		5	35.56	3.00		5	64.60	2.66	
T_2_ *nud* #8–3	20	85.03	4.16	n.s	4	34.20	3.02	n.s	4	80.01	1.71	***
T_2_ *nud* #8–10	40	84.18	5.15	n.s	8	33.05	2.47	n.s	4	76.85	1.28	***

***, significantly different at *p* < 0.001, and *n.s.* not significantly different from DH120366, as determined by Dunnett’s multiple comparison tests

Finally, we conducted grain germination tests in four independent experiments, as good grain germination is crucial for malt production. In the first trial, we conducted the germination test after 4 weeks of after-ripening without dehusking the grain. Under these conditions, the germination rates for *nud* #8–3 and #8–10 (T_3_ grains) were 79% and 82%, respectively, compared to 86% for DH120366 (Table 3). We conducted an additional trial after removing the husks and allowing 5 weeks of after-ripening. The germination rates for *nud* #8–3 and #8–10 were 88% and 98%, respectively, while DH120366 had a germination rate of 90% (Table 3). These differences were not significant, indicating that the *nud* #8–3 and #8–10 mutations did not interfere with germination.

**Table 3:** Grain germination test of the wild type and *nud* mutants

		1st test			2nd test		
	Biological replication	Germination rate	SD		Germination rate	SD	
DH120366	4	0.86	0.04		0.90	0.04	
T_3_ *nud* #8–3	4	0.79	0.07	n.s	0.88	0.15	n.s
T_3_ *nud* #8–10	4	0.82	0.12	n.s	0.98	0.04	n.s

*n.s.* not significantly different from DH120366

## Discussion

Full Pint, one of the parents of the DH120366 line used in this study, is very difficult to transform and is not amenable to *Agrobacterium*-mediated transformation. DH120366 was previously selected by our group from a segregating population derived from a cross between Full Pint and Golden Promise for the presence of the *TFA* alleles from Golden Promise. Therefore, we used DH120366 as the background for our targeted mutagenesis procedure (Hisano et al. 2017). This line has high transformation efficiency (23.7% in previous report) and good potential malting quality, making it a good candidate for the genetic modification of *NUD* via CRISPR/Cas9 to produce naked barley for malting purposes. In the current study, we developed naked barley lines with normal growth parameters beyond their naked grains, which should make them suitable for malting and brewing trials.

The NUD transcription factor regulates lipid biosynthesis; its loss of function disrupts the accumulation of lipids in the cuticle between the lemma and pericarp of barley caryopses, thereby preventing their adhesion (Taketa et al. 2008). While McAllister et al. (2022) measured the abundance of major wax components isolated from flag leaf sheaths and spikes in wax-less mutants, no such information exists for barley lines with naked grains. Although we did not analyze the composition or contents of major lipids in this study, we examined the spikes and did not notice clear visible differences caused by wax deficiency between the wild type and the *nud* mutant lines we generated. At the full ripening stage, the caryopsis was visible between the lemma and pericarp (Fig. 1) in the naked line.

Covered barley is typically used for brewing and distilling, whereas naked barley is utilized for other end-uses (Meints et al. 2021). Introducing the naked barley trait from naturally occurring or induced *nud* mutant lines into malting barley involves crossing, followed by the selection of lines with desirable traits. However, naked barley, which is widely distributed in East Asia, is mainly used for food and contains traits undesirable for brewing, such as high β-glucan levels. Therefore, when introducing the naked caryopsis trait into malting barley, several generations of backcrossing to malting barley cultivars may be required after the initial cross, which is time-consuming and may not entirely remove all genomic regions linked to the naked trait gene. In this study, by modifying only the *NUD* gene, we successfully introduced the naked trait into covered barley while potentially preserving desirable malting and brewing traits. We used a cutting-edge direct breeding technique that leverages the benefits of targeted mutagenesis via CRISPR/Cas9. Such innovative approaches should be further explored.

X-ray-induced naked mutant barley lines showed shorter stems and a lower thousand-grain weight than their parental lines when grown in different locations and over several years (Takahashi et al. 1962). By contrast, the naked barley lines developed in this study should only carry a mutation in *NUD* (introduced via CRISPR/Cas9-based targeted mutagenesis) but should not exhibit other distinct phenotypes, in contrast to mutant lines developed through random mutagenesis. Indeed, we found no significant differences between the wild type and the newly generated *nud* mutants regarding culm length or thousand-grain weight (Table 2, Figure S3). Previous reports have noted that *NUD* is a pleiotropic gene that affects biomass-related traits in barley. For example, Choo et al. (2001) and Barabaschi et al. (2012), upon investigating doubled haploid lines derived from a cross between naked and covered barley cultivars, reported that naked barley progeny had shorter culms and lower thousand-grain weight than covered barley. The authors concluded that the *nud* genotype affected the biomass of these doubled haploid populations, regardless of the genetic background or the original phenotypes of the parental lines. However, the genes *DENSE SPIKE 1* (*DSP1*) and *SHORT AWN 2* (*LKS2*) are located near *NUD* on chromosome 7H and are themselves associated with semi-dwarfism. It is therefore possible that some of the phenotypic differences between the covered and naked barley lines were due to the specific alleles present for these two genes. Notably, DH120366, which was used in the current study, was derived from a cross between two elite cultivars with short stems and may have masked any effect caused by the induced *nud* mutations. In this study, we achieved a 19–24% increase in hectoliter grain weight in the *nud* mutants compared to the wild type (Table 2). This is due to the absence of hulls; similar values were previously reported for naked cultivars (Meints et al. 2015).

Since we grew all barley plants in a growth chamber, we did not obtain enough grains to perform a detailed analysis of malting and brewing traits. However, we investigated how targeted mutagenesis altered important characteristics such as the grain germination rate. We did not observe significant differences in germination rates among the grains from any of the genotypes tested using hand-threshed grains. Machine-threshed grains may have lower germination rates than hand-threshed grains due to damage to the embryo. It will be important to take into consideration the effect of threshing methods on the malting traits related to germination in assessing future mutants.

Further analyses of malting and brewing traits will require field cultivation, which in turn requires confirmation of the absence of the transgene. Therefore, in this study, we carried out basic experiments in anticipation of such assays in the field. We performed comprehensive shotgun sequencing analysis of the genome from the *nud* #8–10 plant, in which we detected no transgene by PCR and *k*-mer analysis (Fig. 2). We conducted a similar analysis on the progenies of the *nud* #8–3 plant backcrossed with DH120366; the *nud* #8–3–7–8 and *nud* #8–3–10–5 plants were free of the transgene, as determined by PCR detection. However, shotgun sequencing of their genomes detected 20–21 bp fragments that were identical to fragments from the T-DNA used for transformation (Figure S4). It is unclear why we detected sequences homologous to T-DNA. The fact that fragments were detected in different locations between *nud* #8–3–7–8 and *nud* #8–3–10–5, even though both lines were derived from *nud* #8–3, suggests that spontaneous mutations in the genome might have accidentally occurred during the process of tissue culture or an error might have occurred during next-generation sequencing. Nonetheless, *nud* #8–10 might be a suitable candidate material to be developed in the future.

In the T_1_ generation, *nud* #8–3 and #8–10 were homozygous for a 13-bp deletion and a 1-bp insertion, respectively, while *nud* #8–8 was heterozygous for the 13-bp deletion, as its other *NUD* copy was not mutated (Table S4). The wild type had also been observed in T_1_ generation. This observation suggests that the original T_0_ *nud* #8 plant was chimeric, rather than just biallelic, at *NUD*. The mutants *nud* #8–3 and #8–10 were derived from different transformation events, and the number and locations of transgenes may differ in each event. We also assessed the possibility of off-target mutations in these *nud* plants, identifying potential off-target sites based on their similarity to the sequence targeted by sgRNA8 (Table S3). Following Sanger sequencing of PCR amplicons covering these potential off-target sites, we detected no mutations in any of these sequences in the *nud* mutants (Figure S5).

In conclusion, we successfully produced naked barley lines from a covered barley line with potential malting quality by targeting the *NUD* gene by targeted mutagenesis. The resulting mutant lines showed little change in traits other than the grain hull trait compared to the parental line, although the volume weight increased due to the absence of a hull. This greater test (volume) weight may be agriculturally advantageous by saving space during the storage and transport of barley grains. We also confirmed the absence of the transgene by PCR genotyping and high-throughput sequencing and of potential off-target mutations by Sanger sequencing; these tests are required before performing field trials of genome-edited crops.

## Data Availability

The accession numbers of short read sequences are SAMD00856266 (DH120366), SAMD00856267 (*nud* #8–3), SAMD00856269 (*nud* #8–3-10–5), SAMD00856268 (*nud* #8–3-7–8), and SAMD00856270 (nud *#*8–10). The accession number for the Bioproject is PRJDB19869.

## Supplementary information

Below is the link to the electronic supplementary material. Supplementary file1 (PDF 4100 KB)
