# Supplementary material for: Rapid development of naked malting barley germplasm through targeted mutagenesis
Source: Mol Breed. 2025 Mar 7;45(3):32. doi: 10.1007/s11032-025-01553-5 (PMC11889295; doi:10.1007/s11032-025-01553-5)
Supplement: Supplementary file 1 — Supplementary file1 (PDF 4100 KB) [file 11032_2025_1553_MOESM1_ESM.pdf]

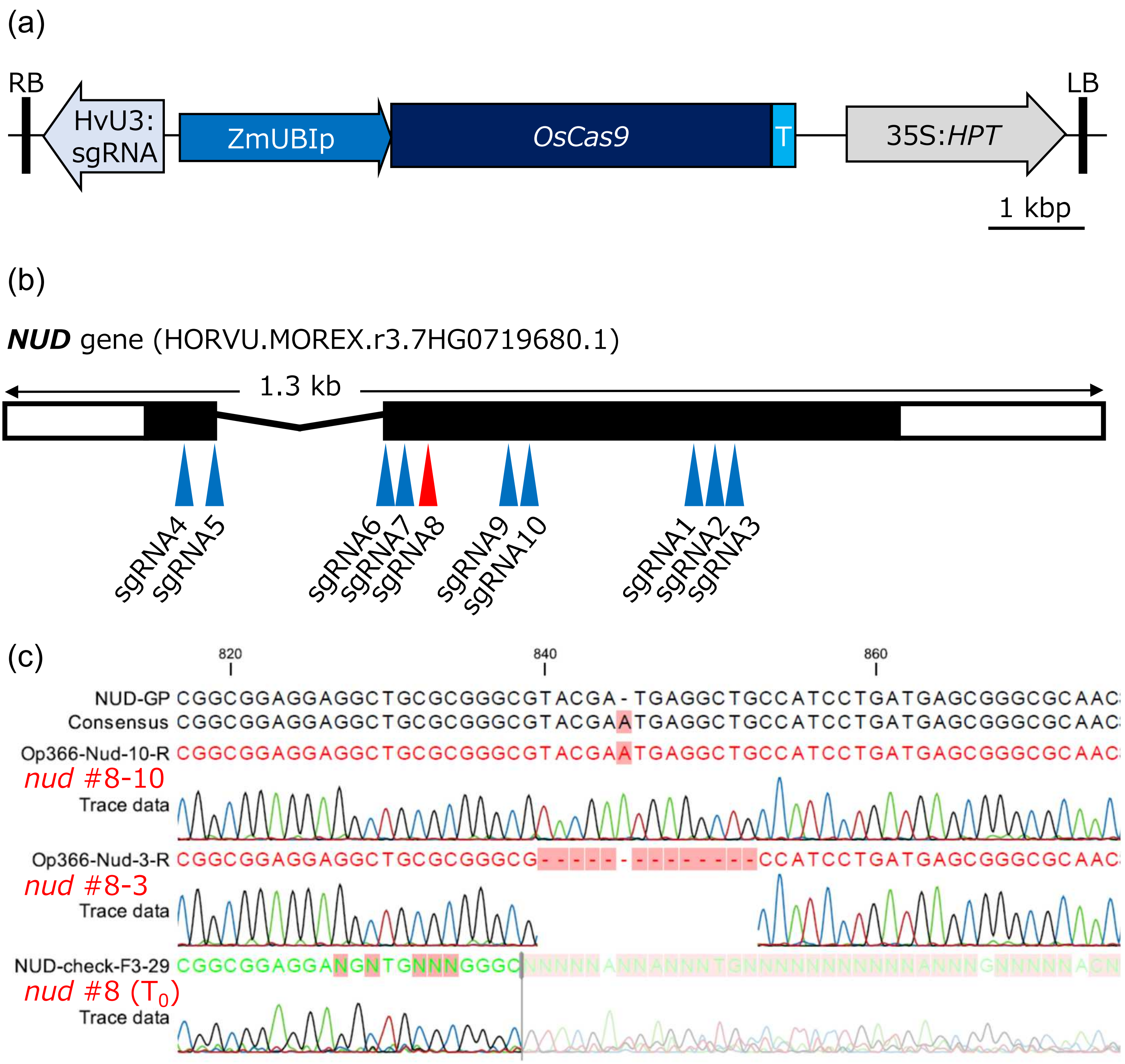

Figure S1. Construction map, sgRNA target position, and mutant sequences.
(a) A construction map used in this study. HvU3; barley *U3* promoter, sgRNA; single guide RNA including target and scaffold sequence, ZmUBIp; the promoter and first intron of maize *ubiquitin* gene, *OsCas9*; rice codon-optimized *Cas9 nuclease* gene, T; pea 3A terminator, 35S; CaMV35S promoter, *HPT*; the *hygromycin phosphotransferase* gene, LB and RB; left and right border region of T-DNA.
(b) Structure of the *NUD* gene and target positions of single guide RNAs (sgRNAs). The positions of the sgRNAs are indicated by arrows. A red arrow indicates the sgRNA8.
(c) Sequence chromatograms around the target site of the sgRNA8 on the *NUD* gene in the mutants, *nud* #8-3, *nud* #8-10, and their ancestor, T_0 *nud* #8.

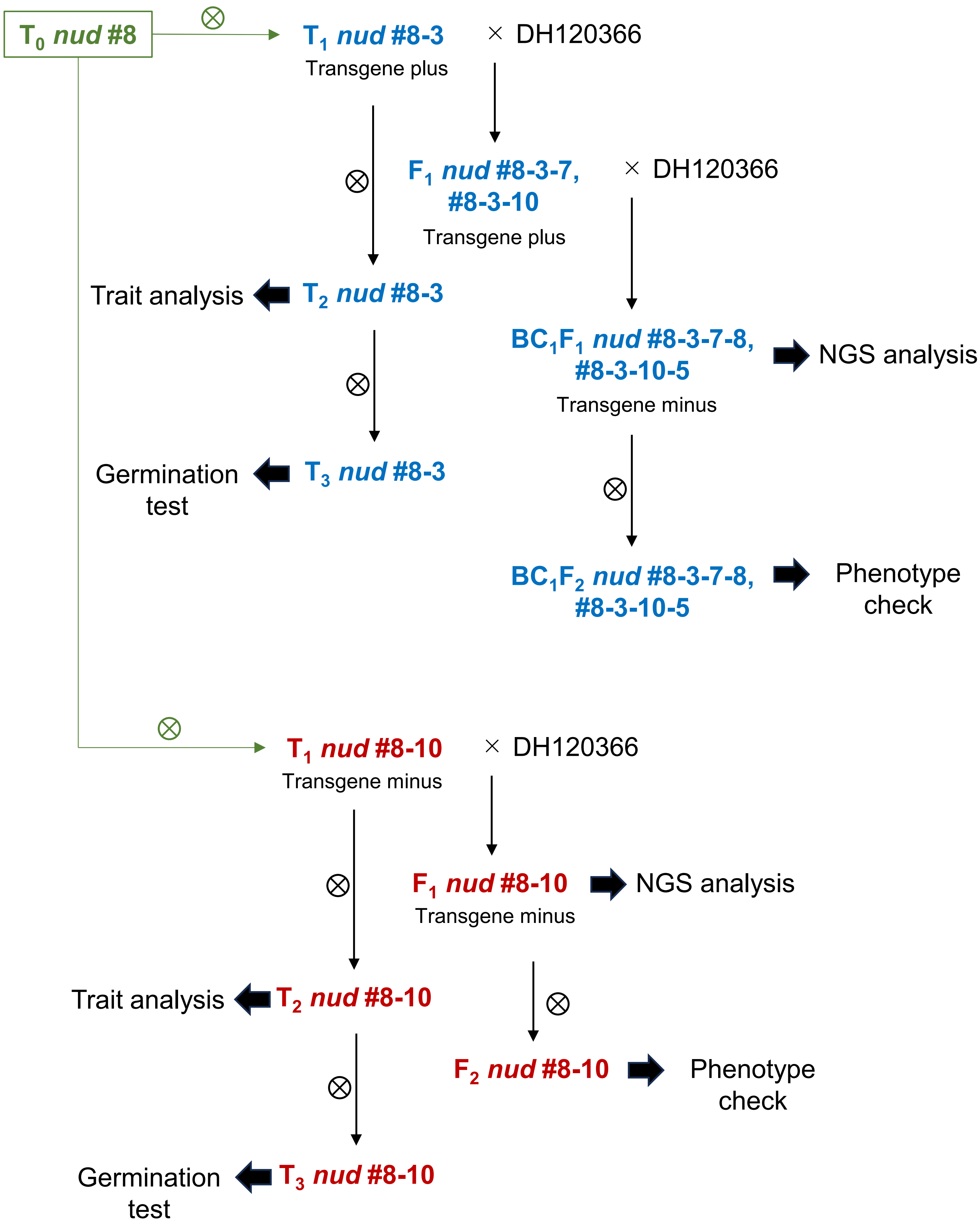

Figure S2. Flowchart of the procedure used to generate plant materials

```
WT          MVQSKKKFRGVRQRHWGSWVSEIRHPLLKRRVWLGTFETAEEAARAYDEA   50
nud #8-10   MVQSKKKFRGVRQRHWGSWVSEIRHPLLKRRVWLGTFETAEEAARAYE*    48
nud #8-3    MVQSKKKFRGVRQRHWGSWVSEIRHPLLKRRVWLGTFETAEEAARAPS*    48
```

The predicted normal amino acid sequence of NUD is 227aa.

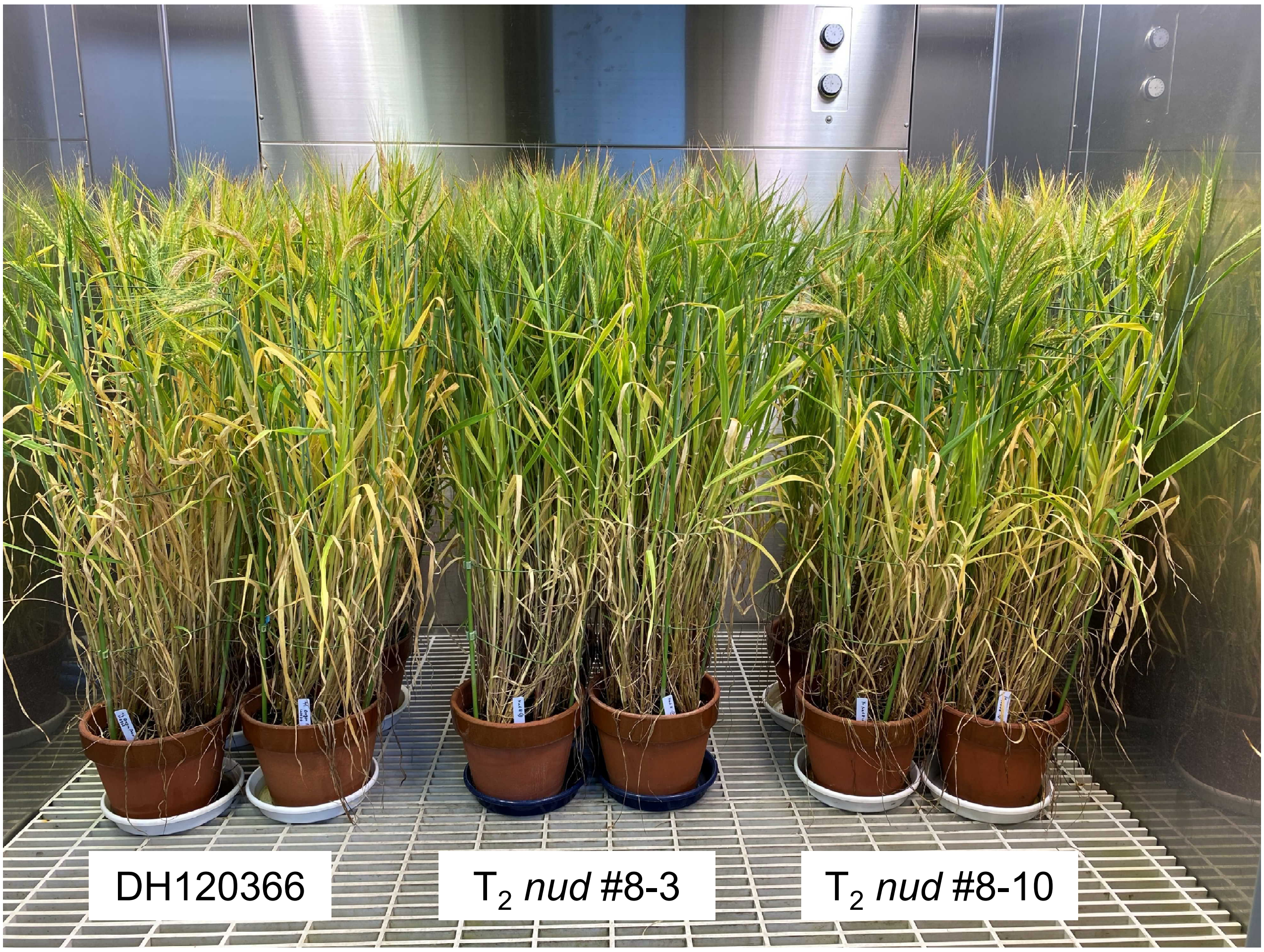

Figure S3. Predicted amino acid sequences of NUD (227 aa) in the *nud* mutants, and representative photograph of wild-type and mutant plants grown in a closed growth chamber

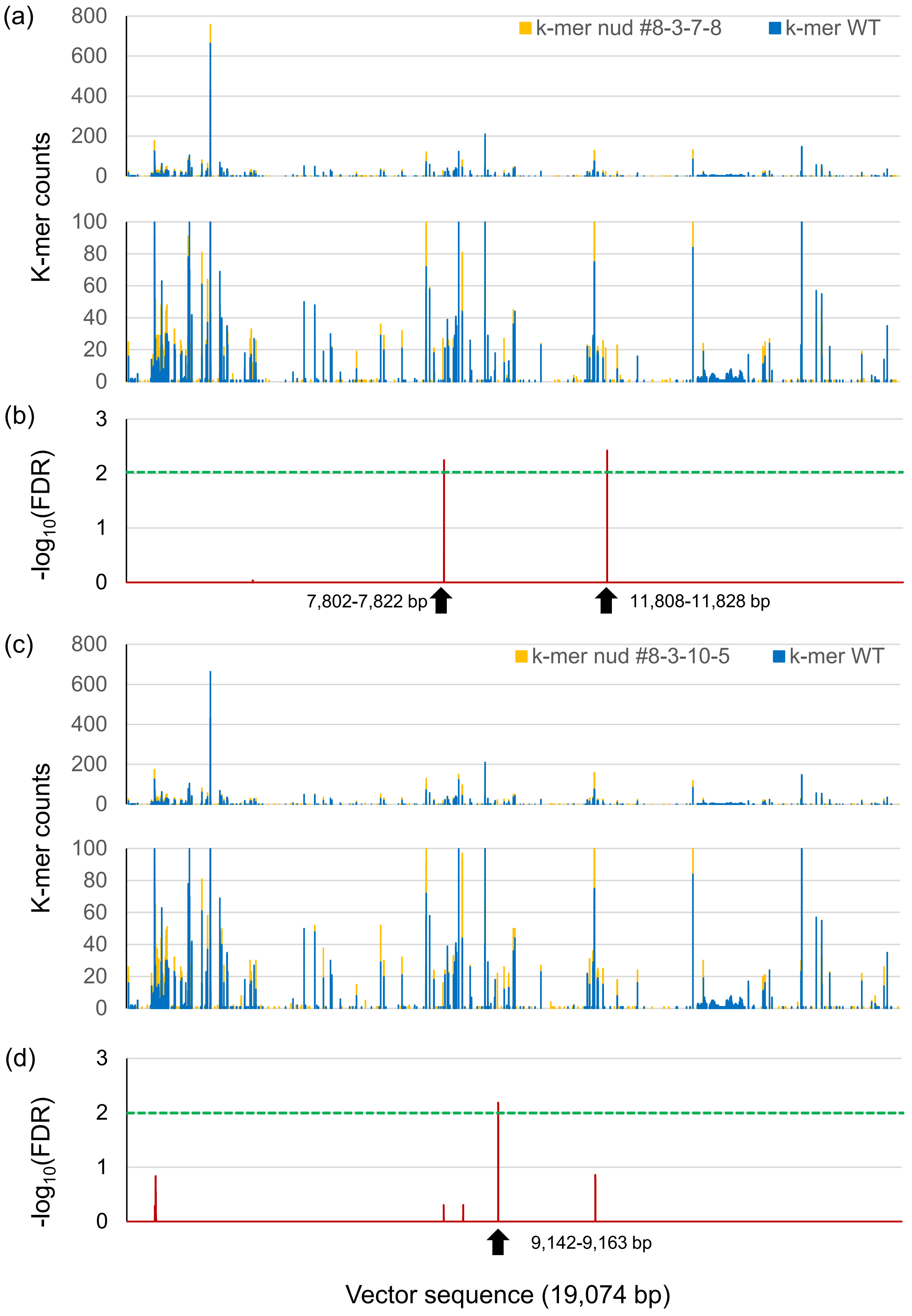
(a)
800
600
400
200
0
100
80
60
40
20
0
K-mer counts
k-mer nud #8-3-7-8
k-mer WT
(b)
3
2
1
0
-log10(FDR)
7,802-7,822 bp
11,808-11,828 bp
(c)
800
600
400
200
0
100
80
60
40
20
0
K-mer counts
k-mer nud #8-3-10-5
k-mer WT
(d)
3
2
1
0
-log10(FDR)
9,142-9,163 bp
Vector sequence (19,074 bp)

Figure S4. Transgene detection in *nud* #8-3-7-8 and *nud* #8-3-10-5 barley plants using the *k*-mer-based method.
(a, c) Numbers of reads counted by the *k*-mer-based method, and **(b, d)** the false discovery rate (FDR)-adjusted *p*-values for potential transgene presence. The *x*-axis represents nucleotide positions covering the vector sequence from 1 bp to 19,074 bp. Top, overall views; bottom, magnified views. Blue indicates read counts for the wild-type control, and yellow represents the mutant **(a)** *nud* #8-3-7-8 and **(c)** *nud* #8-3-10-5. The solid red and dashed green lines correspond to expected values as "$-\log_{10}$(FDR)" and the 1% significance threshold, respectively, for **(a)** *nud* #8-3-7-8 and **(c)** *nud* #8-3-10-5.

Figure S5

Hisano et al.

(Potential off-target site 1)

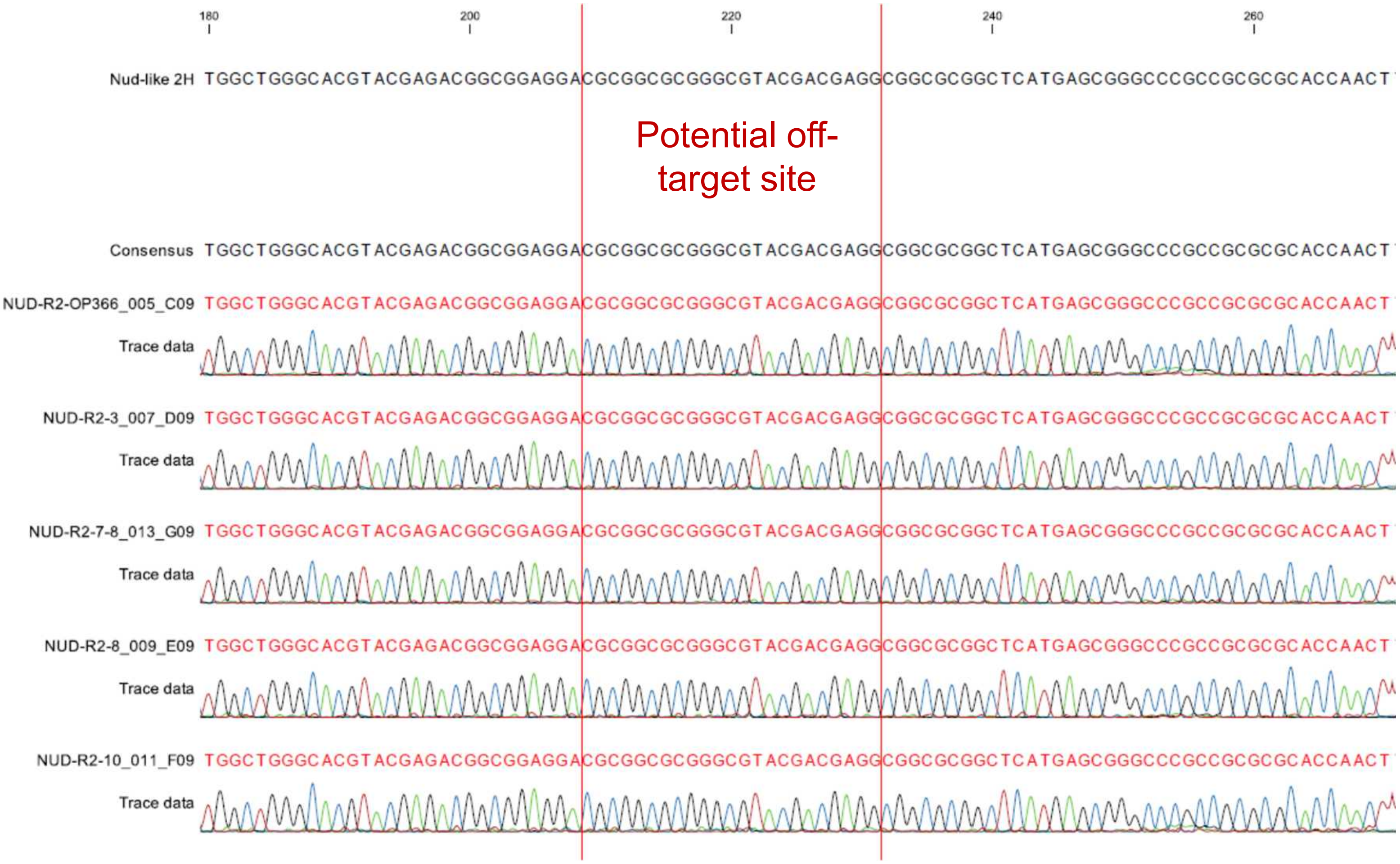

(Potential off-target site 2)

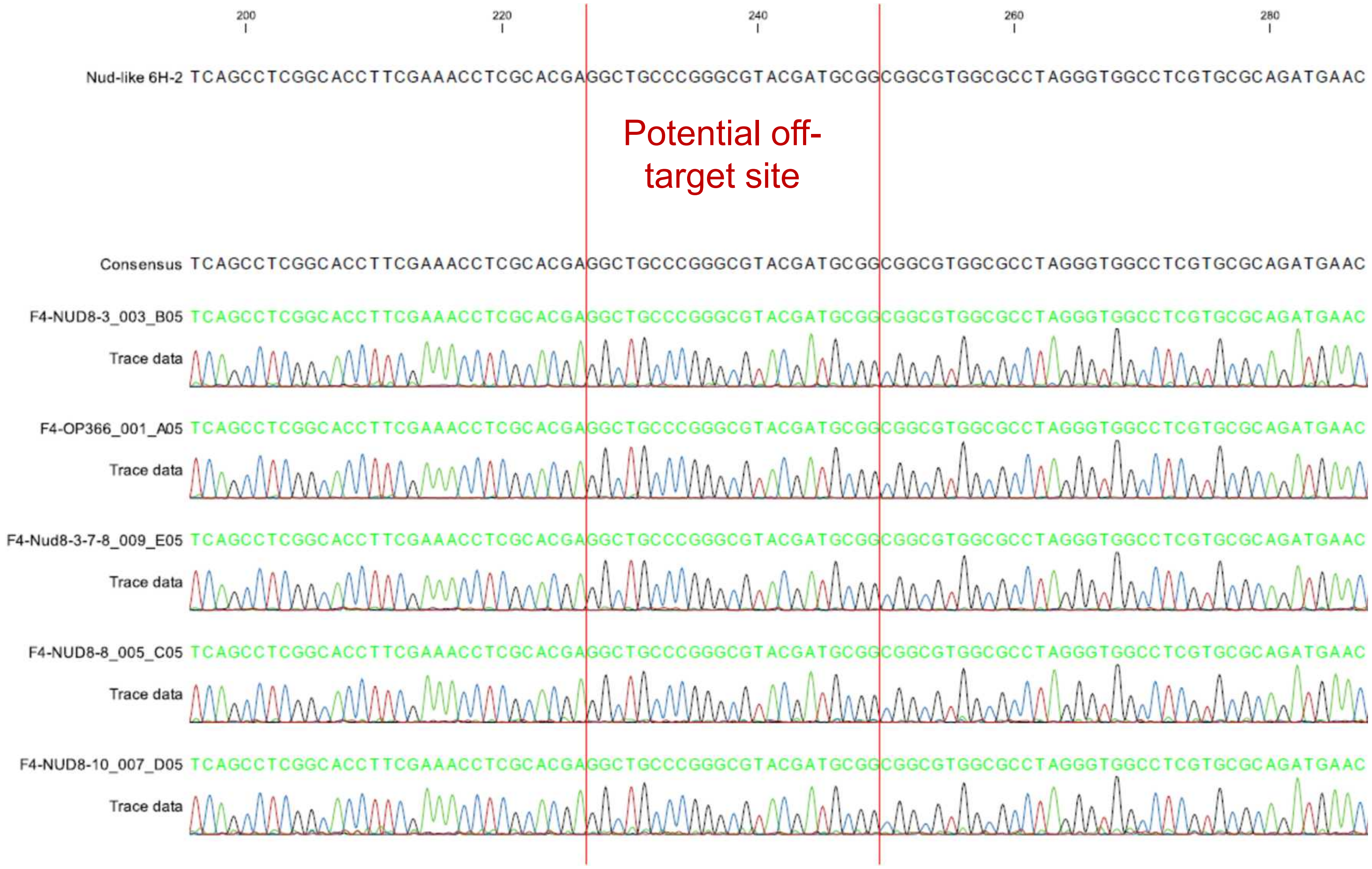

(Potential off-target site 3)

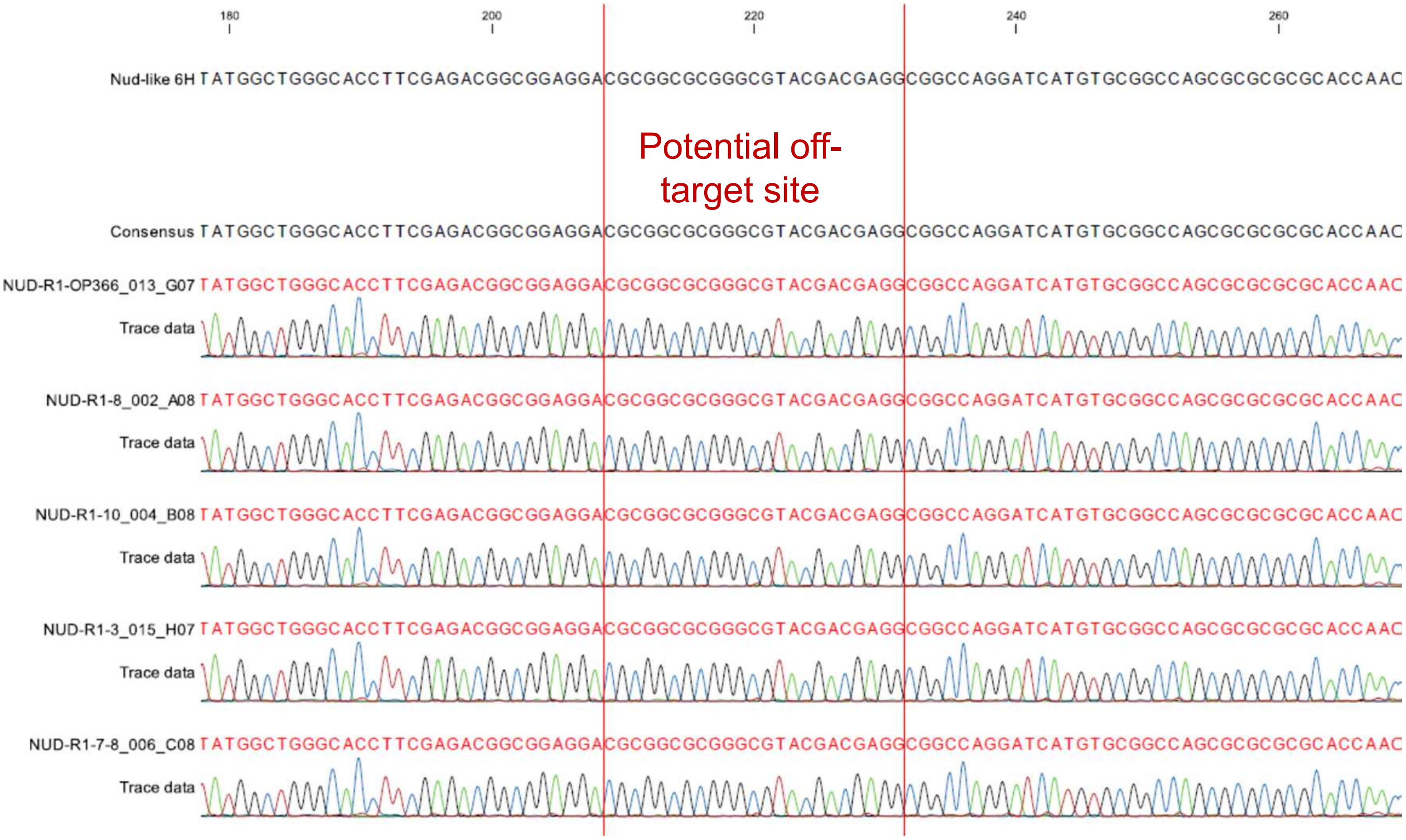

(Potential off-target site 4)

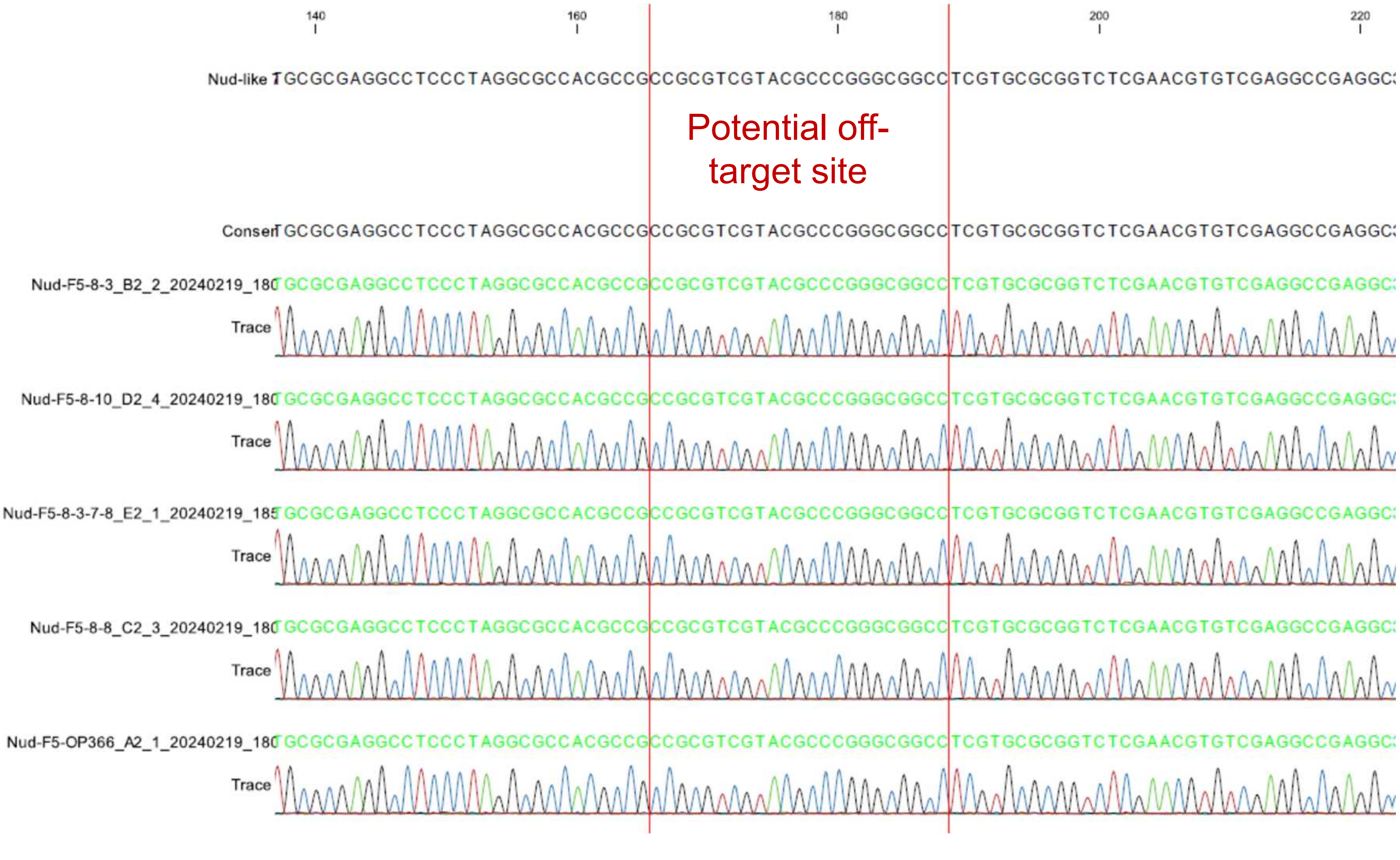

(Potential off-target site 5)

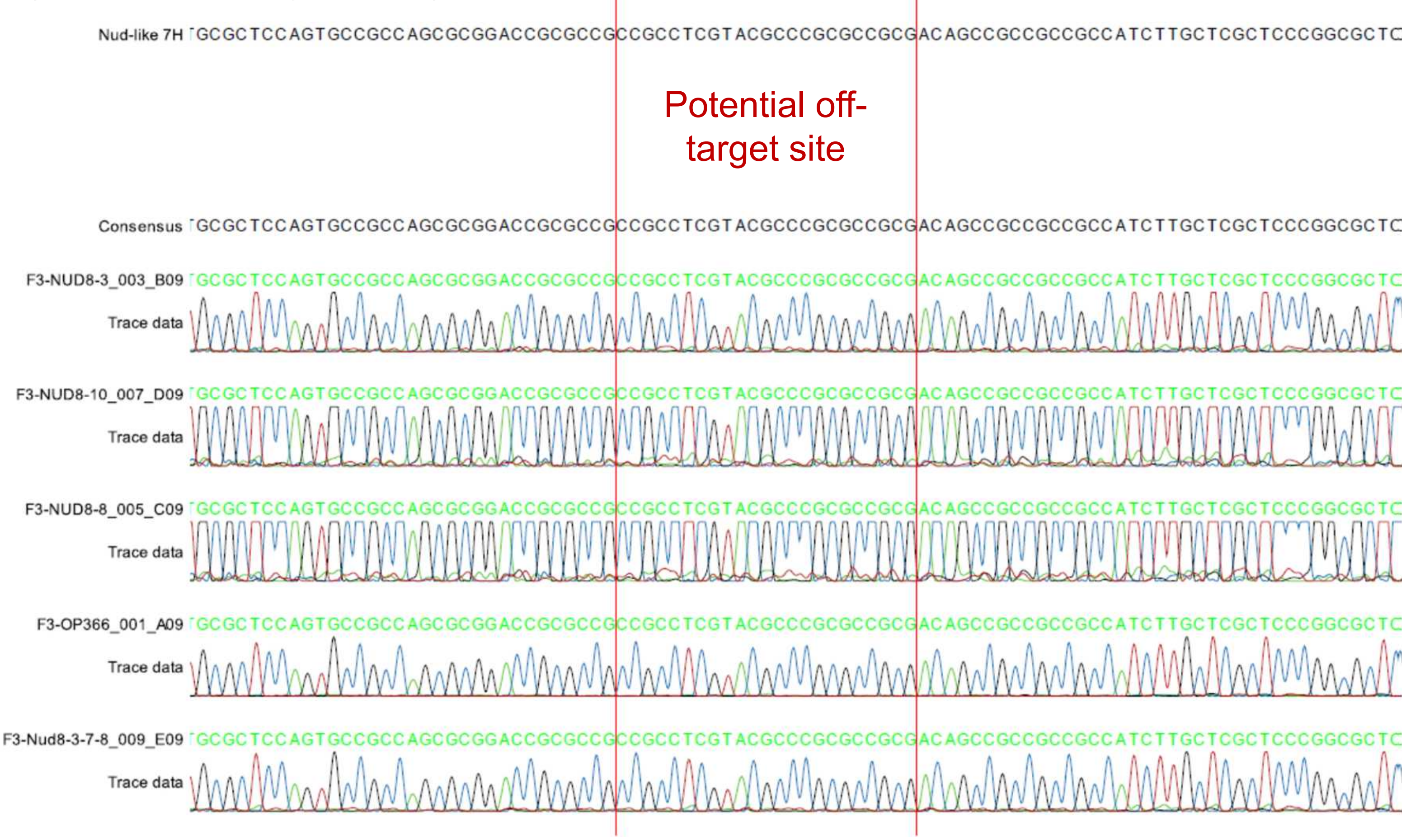

(Potential off-target site 6)

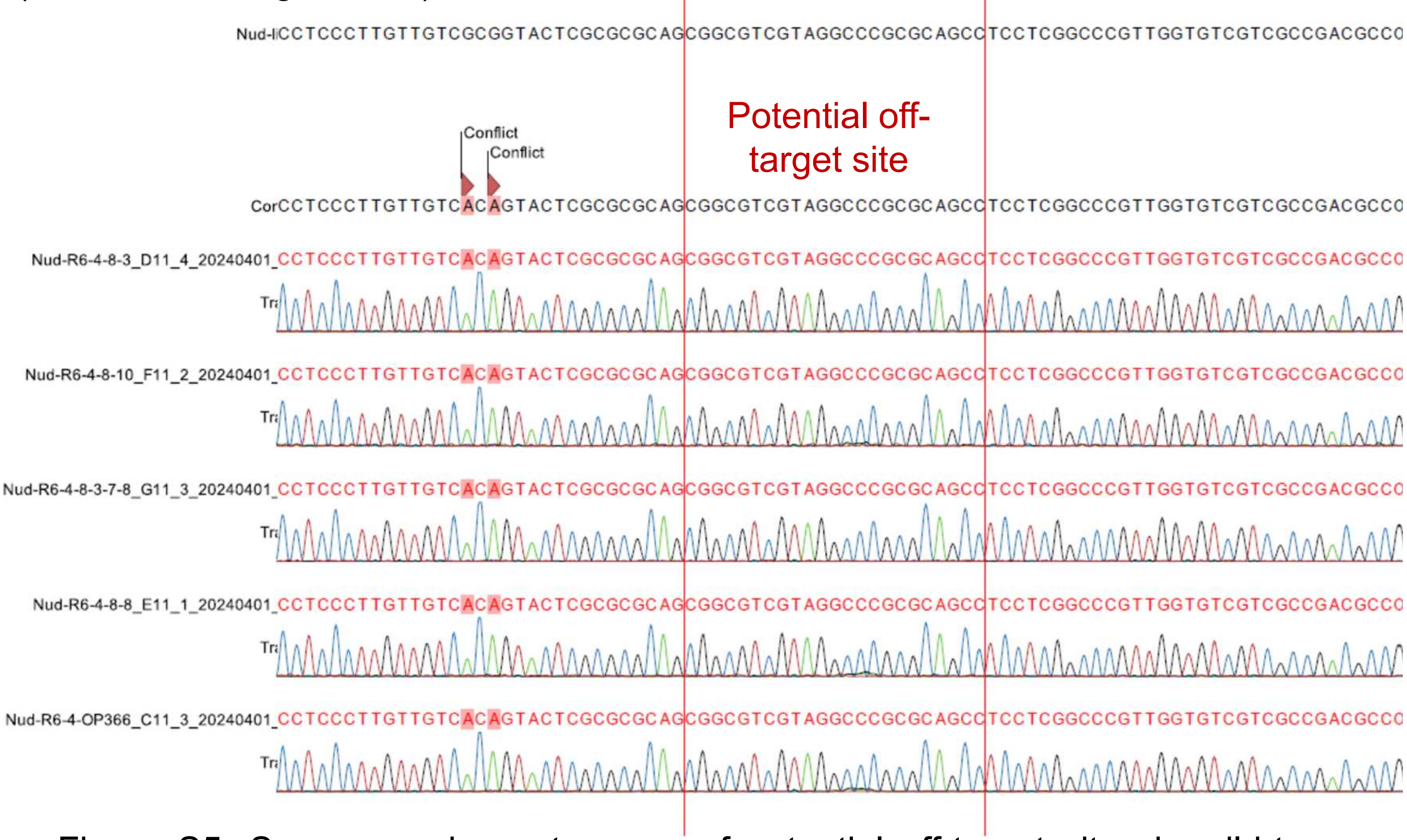

Figure S5. Sequence chromatograms of potential off-target sites in wild-type and *nud* plants

Table S1. Malting quality of Oregon Promise doubled haploid progeny grown in 2015 (modified from Herb et al. (2017))

Field location	Genotype	AA	BG	DP	FAN	MC	ME	SP	S/T	TP	VC
	**120366**	64.3	62	113	179	1.87	78.89	4.24	42.69	9.94	1.5
	120536	47.1	200	100	101	1.41	77.52	3.28	29.04	11.29	1.59
Corvallis OR, USA	120543	38.9	347	75	111	1.25	76.99	1.65	16.13	10.22	1.71
	Golden Promise	46.5	372	106	123	1.46	77.49	3.44	35.03	9.83	1.64
	Full Pint	92.7	293	148	191	1.72	78.78	4.75	42.33	11.23	1.62
	**120366**	44.3	264	130	121	1.26	75.34	3.53	27.46	12.87	1.57
	120536	38.6	842	91	103	1.4	75.09	3.56	26.21	13.59	1.81
Corvallis OR, USA	120543	72.3	100	177	241	1.65	77.55	5.07	42.51	11.91	1.5
	Golden Promise	37.9	599	89	102	1.77	77.76	2.88	27.91	10.34	1.62
	Full Pint	79.5	370	150	137	1.42	79.1	4.44	39.05	11.37	1.45
	**120366**	52	206	156	124	1.57	77.08	5.12	38.34	13.35	1.61
	120536	33	555	136	112	1.51	74.98	3.96	27.91	14.19	1.78
Madras OR, USA	120543	39	279	126	200	1.17	73.92	4.07	31.11	13.08	1.71
	Golden Promise	44	491	162	122	1.26	74.69	4.24	32.44	13.06	1.71
	Full Pint	78	402	170	175	1.23	77.34	4.79	40.96	11.7	1.64

AA: alpha-amylase; BG: beta-glucan; DP: diastatic power; ME: MC: malt color; malt extract; SP: soluble protein; S/T: Kolbach Index; TP: total protein; FAN: free amino nitrogen; VC: viscosity

Table S2. The target site of each single guide RNA (sgRNA) used to target NUD, and DNA oligos used in this study

gRNA	Target sequence	DNA oligo for construction
gRNA1	GTGCCCGTGCCGACTCCAGC	5'- GTTGGTGCCCGTGCCGACTCCAGC -3'<br>5'- AAACGCTGGAGTCGGCACGGGCAC -3'
gRNA2	CTCGACGGTCATGACCCAGC	5'- GTTGCTCGACGGTCATGACCCAGC -3'<br>5'- AAACGCTGGGTCATGACCGTCGAG -3'
gRNA3	CGGCTCCTTGTTGAGCTCGA	5'- GTTGCGGCTCCTTGTTGAGCTCGA -3'<br>5'- AAACTCGAGCTCAACAAGGAGCCG -3'
gRNA4	CGGAGACCCAGGAGCCCCAG	5'- GTTGCGGAGACCCAGGAGCCCCAG -3'<br>5'- AAACCTGGGGCTCCTGGGTCTCCG -3'
gRNA5	TAGCGAGAGGCTTACAGGAG	5'- GTTGTAGCGAGAGGCTTACAGGAG -3'<br>5'- AAACCTCCTGTAAGCCTCTCGCTA -3'
gRNA6	TATGGTTTACAGGAAGAGGA	5'- GTTGTATGGTTTACAGGAAGAGGA -3'<br>5'- AAACTCCTCTTCCTGTAAACCATA -3'
gRNA7	GTTGGGCACCTTTGAGACGG	5'- GTTGGTTGGGCACCTTTGAGACGG -3'<br>5'- AAACCCGTCTCAAAGGTGCCCAAC -3'
gRNA8	GGCTGCGCGGGCGTACGATG	5'- GTTGGGCTGCGCGGGCGTACGATG -3'<br>5'- AAACCATCGTACGCCCGCGCAGCC -3'
gRNA9	GTCCCGTGCTGCTGCTGCTG	5'- GTTGGTCCCGTGCTGCTGCTGCTG -3'<br>5'- AAACCAGCAGCAGCAGCACGGGAC -3'
gRNA10	GCAGCAGCACGGGACATTCG	5'- GTTGGCAGCAGCACGGGACATTCG -3'<br>5'- AAACCGAATGTCCCGTGCTGCTGC -3'

Table S3. Sequence of the NUD gene and potential off-target sites

		Reference	Golden Promise v1				Sequencing primers
#	Sequence	Chr	Start	End	Strand	Mismatch	Forward/Reverse
(1)	cGCgGCGCGGGCGTACGAcG**AGG**	2H	584122040	584122062	+	3	5'- CTCATGTACGTCCACGAACTGTAG -3'<br>5'- CTTGGTCGCCTCGACGTTCTGCAC -3'
(2)	GGCTGCcCGGGCGTACGATG**CGG**	6H	269931499	269931521	+	1	5'- GTCACATCTTGGGCGTTACACATG -3'<br>5'- CTTGTCATCCTCTCCGCCCATAAG -3'
(3)	cGCgGCGCGGGCGTACGAcG**AGG**	6H	497863114	497863092	-	3	5'- CCTGTGCGTACCTACCTGTCTACC -3'<br>5'- CCTGCACGGACGCCATGTTGAACC -3'
(4)	GGCcGCcCGGGCGTACGAcG**CGG**	7H	243168278	243168256	-	3	5'- CTCGTCCTCCTCGACGATGAGGAG -3'<br>5'- CTCGTGAGGGCCTTTCCTTGAGTG -3'
(5)	cGCgGCGCGGGCGTACGAgG**CGG**	7H	465047914	465047892	-	3	5'- AGATCTACTGGCAACTGCTACCAG -3'<br>5'- AACCCACGATGCCTACCAGTTGAG -3'
NUD	GGCTGCGCGGGCGTACGATG**AGG**	7H	486189149	486189127	+	0	5'- GAGAATCTCGCTCGCTCTG -3'<br>5'- GATCTGTGACAGGCTGCTG -3'
(6)	GGCTGCGCGGGCcTACGAcG**CcG**	7H	557157348	557157326	-	3	5'- GCACGGAGTCGAAGGGTGGAAGGC -3'<br>5'- CAACTTGATACGCACACAACCATC -3'

#: Potential off-target site number in Figure S4

Table S4. Analysis of transgene and target sequences in T_1 plants

ID	Caryopsis phenotype	*NUD* gene	*HPT* gene
nud #8-1	Naked	WT	+
nud #8-2	Naked	WT	+
nud #8-3	Naked	13-bp deletion (homo)	+
nud #8-4	Naked	Mosaic	+
nud #8-5	Naked	N.A.	N.A.
nud #8-6	Naked	WT	+
nud #8-7	Naked	WT	+
nud #8-8	Naked	13-bp deletion (hetero)	+
nud #8-9	Naked	WT	+
nud #8-10	Naked	1-bp insertion (homo)	-
nud #8-11	Hulled	WT	+
nud #8-12	Hulled	WT	-
nud #8-13	Hulled	WT	+
nud #8-14	Hulled	WT	+
nud #8-15	Hulled	WT	+
nud #8-16	Hulled	WT	+
nud #8-17	Hulled	WT	+
nud #8-18	Hulled	WT	+
nud #8-19	Hulled	WT	+
nud #8-20	Hulled	WT	+

**nud* #8-5 did not germinate. N.A.; Not analyzed.

Table S5. Analysis of transgene and target sequence in *nud* #8-3 x DH120366 BC_1F_1 plants

	HPT gene	*Cas9* gene	*NUD* gene
nud #8-3-7-1	-	-	WT
nud #8-3-7-2	+	+	13-bp deletion (hetero)
nud #8-3-7-3	+	+	13-bp deletion (hetero)
nud #8-3-7-4	-	-	WT
nud #8-3-7-5	+	+	13-bp deletion (hetero)
nud #8-3-7-6	+	+	Hetero
nud #8-3-7-7	+	+	Hetero
***nud* #8-3-7-8**	**-**	**-**	**Hetero**
nud #8-3-7-9	+	+	13-bp deletion (hetero)
nud #8-3-7-10	-	-	WT
nud #8-3-7-11	+	+	13-bp deletion (hetero)
nud #8-3-7-12	-	-	WT
nud #8-3-7-13	+	+	13-bp deletion (hetero)
nud #8-3-7-14	+	+	Hetero
nud #8-3-7-15	+	+	13-bp deletion (hetero)
nud #8-3-7-16	+	+	Hetero
nud #8-3-7-17	+	+	WT
nud #8-3-7-18	+	+	Hetero
nud #8-3-7-19	+	+	Hetero
nud #8-3-7-20	-	-	WT
nud #8-3-10-1	+	+	Hetero
nud #8-3-10-2	+	+	Hetero
nud #8-3-10-3	+	+	Hetero
nud #8-3-10-4	+	+	Hetero
***nud* #8-3-10-5**	**-**	**-**	**Hetero**
nud #8-3-10-6	+	+	13-bp deletion (hetero)
nud #8-3-10-7	-	-	WT
nud #8-3-10-8	+	+	Hetero
nud #8-3-10-9	-	-	WT
nud #8-3-10-10	-	-	WT
nud #8-3-10-11	+	+	Hetero

WT; wild type

Hetero; heterozygous mutations

Table S6. Summary of high-throughput sequencing data to detect the transgene

Sample	Note	Reads	Total length 1 (bp)	Total length 2 (bp)	Total length (bp)
DH120366	negative control	388,678,289	58,301,743,350	58,502,756,140	116,804,499,490
nud #8-3	positive control (T_1)	434,157,226	65,123,583,900	69,465,156,160	134,588,740,060
nud #8-3-7-8	BC_1F_1	438,489,469	64,273,420,350	68,558,315,040	132,831,735,390
nud #8-3-10-5	BC_1F_1	447,124,752	67,068,712,800	71,539,960,320	138,608,673,120
nud #8-10	F_1	393,200,294	58,980,044,100	61,384,020,830	120,364,064,930
